# From the Macro to the Micro: Gel Mapping to Differentiate between Sporozoites of Two Immunologically Distinct Strains of *Eimeria maxima* (Strains M6 and Guelph)

**DOI:** 10.1371/journal.pone.0143232

**Published:** 2015-12-07

**Authors:** Saeed El-Ashram, Qing Yin, Hongbin Liu, Ibrahim Al Nasr, Xianyong Liu, Xun Suo, John Barta

**Affiliations:** 1 State Key Laboratory for Agrobiotechnology, China Agricultural University, Beijing, 100193, China; 2 National Animal Protozoa Laboratory & College of Veterinary Medicine, China Agricultural University, Beijing, China; 3 Faculty of Science, Kafr El-Sheikh University, Kafr El-Sheikh, Egypt; 4 University of Guelph, Guelph, Ontario, Canada; 5 Department of Pharmacology, Hebei North University, Zhangjiakou, Hebei, China; 6 College of Applied Health Sciences in arras, Qassim, Qassim University, Saudi Arabia; Instituto Butantan, BRAZIL

## Abstract

Two immunologically distinct strains of *E*. *maxima* were examined in this study: the M6 strain and the Guelph strain. The differential expression between the sporozoites of the two strains of *E*. *maxima* was determined by image analysis of 100 μg of protein from each strain separated by standard one- and conventional two-dimensional polyacrylamide gel electrophoresis. In addition to differences in both molecular weight and the electrophoretic mobility, differences in the intensity of polypeptide bands for example, GS 136.4 and M6 169 were explored. Pooled gels were prepared from each strain. A representative 2D-PAGE gel spanning a non-linear pH range of 3–10 of *E*. *maxima* strain M6 consisted of approximately 694 polypeptide spots with about 67 (9.6%) of the polypeptide spots being unique relative to the other strain. *E*. *maxima* strain GS had about 696 discernable polypeptide spots with 69 spots (9.9%) that differed from those of the M6 strain. In-depth characterization of the variable polypeptide spots; unique polypeptide spots (absence or presence) and shared polypeptide spots with modifications may lead to novel vaccine target in the form of multi-component, multi-stage, multi-immunovariant strains, multi-species subunit vaccine, and diagnostic probe for *E*. *maxima*.

## Introduction

*Eimeria* species are obligate intracellular apicomplexan protistan parasites. They are the major cause of chicken coccidiosis, a disease that leads to economic losses in livestock industries, particularly poultry due to intensive rearing conditions. Diarrhea, weight loss, haemorrhage, general weakness, and death are some of the severe clinical signs of infection. The life cycle of *Eimeria* consists of three stages sporogony (exogenous stage), gamogony, and merogony (endogenous stages). It is one host life cycle, which starts with the ingestion of the sporulated oocysts. Upon ingestion, excystation occurs under the influence of CO_2_, bile salts and trypsin. The liberated sporozoites invade the intestinal epithelial cells (IECs) and intestinal intraepithelial lymphocytes (IELs). The latter is used as translocation vehicle to reach to the predilection sites (i.e. the proliferative compartment; the crypt) then the trophozoites undergo three to five merogonic cycles before differentiation to male and female gametes. After fertilization, the unsporulated oocysts pass out with feces, and then undergo sporulation in moisture, and oxygen-rich environment. *Eimeria* species have evolved in a way to invade the intestinal epithelial cells from duodenal loop to cecal pouches, therefore, *Eimeria* spp. are both host and site-specific. *E*. *maxima* or mid-gut coccidiosis is one of the migratory species of *Eimeria*. Intensive rearing conditions, anti-coccidial feed additives, and live oocyst vaccination brought *Eimeria* species under selection pressure that led to the generation of immunologically distinct species and even strains. Two laboratory strains of *E*. *maxima* are compared in this study; the M6 strain was isolated from a commercial broiler house in Florida, USA, in 1995 and subsequently cloned via single oocyst infection. A second strain, designed the Guelph strain, was isolated from chickens in 1973 from Ontario, Canada, and subsequently cloned via single oocyst infection. Both strains have been maintained by cryo-preservation as sporocysts in liquid nitrogen, and propagated in SPF birds as needed to minimize the number of passages since their original isolations. From the biological point of view, the phenotypic criteria, the chemoprophylactic and immunoprophylactic targets are determined by the proteins expressed by each strain. The entire collection of sporozoite proteins is called sporozoite proteome. Two- dimensional polyacrylamide gel electrophoresis (2D-PAGE) has been the core analytical tool for proteomics since its introduction [1]. This high-resolution technique for separating a complex mixture of proteins has been applied to coccidial protein characterization [2], for establishing of a reference map for *Eimeria tenella* sporozoites [3] and for studying purified rhoptry proteins [4]. The same technique has been used widely in the examination of other apicomplexan parasites such as *Plasmodium falciparum* [5–7] *Toxoplasma gondii* [8–10] and *Cryptosporidium* species [11]. However, comparisons of total sporozoite proteins between strains of a single *Eimeria* species have not been reported until now. In this paper we explore the differences between two immunologically distinct strains of *E*. *maxima;* Guelph and M6 strains, by comparing the polypeptide spots of their sporozoites using one- and two-dimensional sodium dodecyl sulphate polyacrylamide gel electrophoresis (2D SDS-PAGE) to enhance our understanding of the previously demonstrated strain-specific nature of the immune responses of chickens to these strains.

## Materials and Methods

### Ethics Statement

This research has received clearance from the University of Guelph Research Ethics Board as consistent with the standards of the Tri-Council Policy Statement for research involving animals. All experimental procedures were specifically approved by the University of Guelph Research Ethics Board.

### Chickens

Three-week-old Barred Rock Chickens were used for oocyst propagation of two *E*. *maxima* strains. The climatic conditions, lighting program, and chicken fodder and water were manually-operated and the chickens were cared for in agreement with the approved guidelines of the Institutional Animal Care and Committee of Guelph University. A total of twenty; 15-day-old chickens were randomly allocated into two groups (i.e. M6- and GS-infected groups) each consisting of 10 birds in a separate room to avoid cross-contamination. The chickens were housed in isolators with external dimensions (2200*850*1800mm) for the entire experiment, with food and tap water provided *ad libitum*. Chickens were exposed to a 12 h/12 h light/darkness regimen at 25°c. The chickens were not abstained from food and water before CO_2_ euthanasia. All efforts were made to minimize animal suffering.

### Parasite strains

Two strains of *E*. *maxima* were compared in this study.

#### *E*. *maxima* Guelph strain (GS)

*E*. *maxima* GS is a single oocyst-derived strain of *E*. *maxima* that was originally isolated from litter samples obtained from a commercial broiler house in Ontario in 1973. Since the initial isolation and subsequent single oocyst cloning, the strain has been maintained at the Ontario Veterinary College, Guelph, Canada by passage through SPF chickens as required. The strain has been stored for longer periods as cryopreserved sporocysts held in liquid nitrogen.

#### *E*. *maxima* M6

This single sporocyst-derived (i.e. genetically clonal) strain of *E*. *maxima* was generated from *E*. *maxima;* Florida strain (FL) [12]. *E*. *maxima* FL was isolated from litter samples during the mid-1990’s from a commercial broiler house in Florida, USA.

### Parasite propagation and sporozoite isolation

#### Oocyst propagation

Parasites were propagated in specific pathogen free (SPF) Barred Rock Chickens (Shaver Strain) according to experimental requirements. Sporulated oocysts were inoculated into SPF chickens by oral gavage, and feces were collected at 7–9 days post-inoculation from which oocysts were isolated by fecal flotation [13]. Briefly, feces containing unsporulated oocysts were mixed thoroughly with saturated sodium chloride (aqueous) in a blender. The mixture was poured through a 1.5 mm mesh size screen to remove large pieces of debris and the filtrate was centrifuged at 1500×g for 15 min. The pellet was decanted, the top layer containing oocysts was diluted at least 10-fold in distilled water, centrifuged as before and the resulting pellet was resuspended in 2.5% potassium dichromate (w/v, aqueous). The partially purified oocysts were sporulated by agitation in Erlenmeyer flasks covered with a perforated lid on a rotary shaker at 26°C for approximately 5 days. Sporulated oocysts required for sporozoite isolation were stored at 4°C for no more than 4 weeks or used within 6 months as inoculum for further vivo propagation in SPF chickens.

#### Sporozoite isolation

Sporozoites destined for protein analyses were purified from surface sterilized, sporulated oocysts as follows. Partially purified sporulated oocysts (see above) were pelleted by centrifugation and resuspended in 4 to 5 volumes of ice-cold household bleach (sodium hypochlorite, ~5% w/v aqueous) and placed on ice for 10 minutes with occasional shaking. A small amount of distilled water (1–2 ml) was carefully layered onto the top of the oocysts suspended in bleach and the tube was loaded into a swinging bucket centrifuge without agitation and centrifuged at 1500×g for 15 min without rotor braking. The upper ¾ of the contents of the centrifuge tube was collected and diluted at least 10-fold in phosphate-buffered saline (PBS; pH 7.2). The oocysts were washed by repeated centrifugation and resuspension in PBS until traces of bleach odor were removed. The oocyst walls of these highly purified oocysts were broken using a Mickle disintegrator (Brinkman, Westbury, New York), and the sporocysts were isolated by filtration through Nitex™ screen-printing cloth with a 15μm pore size. Excystation of the purified sporocysts found in the filtrate was accomplished by incubation in PBS containing 5% (v/v) chicken bile and 0.25% (w/v) porcine trypsin (Sigma-Aldrich). After excystation, freed viable sporozoites were passed through a 6μm Nitex™ screen-printing cloth [14]. To remove the excystation solution, the sporozoites from the same batch of the samples were washed repeatedly in PBS (pH 7.2) or Tris buffer (pH 9.6) as required. The sporozoites were pelleted by centrifugation, and excess buffer was removed by a micropipette. The sporozoite pellets were kept aliquots at -80°C for experimental use.

### Sporozoite preparation for one-and two-dimensional gel electrophoresis

The protein preparation was performed according to [10]. Protein was prepared by rapid freezing and thawing 3 times using liquid nitrogen to disrupt sporozoites in lysis buffer containing 7 M urea, 2 M Thiourea, 4%(w/v) CHAPS, 1%(w/v) DTT, 1 mM PMSF and 0.5%(v/v) IPG buffer pH (3–10) dissolved in 40 mM Tris-base pH 9.6. The concentration of protein was determined using a 2-D Quant Kit (Amersham, Uppsala, Sweden) and used at a concentration of 100μg for the one-and two-dimensional gel electrophoresis.

#### One-dimensional polyacrylamide gel electrophoresis

The protein in lysis buffer was mixed with loading buffer (0.5 M Tris-HCl, pH 6.8, 20% (V/V) Glycerol, 10% SDS, 10% β-mercaptoethanol, 80μl% (V/V) Pyronin (2mg/ml) at a ratio 1:1 and then boiled for 5 minutes before loading. The protein was separated using gradient gels 5% to 20% according to the manufacturer’s instructions (BioRad, CA, USA) at a constant current of 30mA for 30 min followed by 50mA for the remainder of the electrophoresis. The running gels were stained using 0.1% Coomassie brilliant blue R-250 in 50% methanol and 10% glacial acetic acid for 30–60 min, and then destained in 10% and 7% glacial acetic acid.

#### Two-dimensional polyacrylamide gel electrophoresis

Two-dimensional polyacrylamide gel electrophoresis (2D-PAGE) of sporozoite proteins was conducted according to the procedure of [10] as follows: Sporozoite antigens of two *E*. *maxima* strains were subjected to nonlinear immobilized pH gradient strips (IPG) (Immobiline DryStrip, pH 3–10 nonlinear, 13cm, Amersham, Uppsala, Sweden) using the Ettan IPGphor Isoelectric Focusing system (Amersham). Nonlinear IPG strips (pH 3–10) and 13cm in length were rehydrated overnight with 50 μl of lysis buffer containing 100μg protein mixed with 200 μl of rehydration sample buffer (6M Urea; 2M Thiourea; 4%CHAPS;); 65mM DTT; 0.5% IPG (Amersham) and 0.04% w/v bromophenol blue in 40mM Tris-base (pH 9.6)). Proteins were separated according to their isoelectric charge at 500 V for 1 hr, 1000 V for 1hr and 4500 V for 10 hrs at a constant temperature of 20°C. IPG strips were stored at -20°C until use.The IPG strips containing focused sporozoite proteins were equilibrated for 1 hr in a reducing buffer (6M Urea in glycerol (87% v/v), 64.8 mM DTT, SDS (2% w/v), Bromophenol blue (0.04% v/v of 1.5M Tris–HCl, pH 8.8), and then followed by 1 hr in an alkylating buffer (6M Urea, 87%v/v Glycerol, 135 mM iodoacetamide, 2%w/v SDS, 0.04% Bromophenol blue and 1.5 M Tris–HCl pH 8.8). The equilibrated IPG sample strips were subjected to 12.5% vertical SDS-PAGE after embedding in a 0.5% w/v agarose stacking buffer overnight at 30 mA. With all protein samples, broad range molecular weight protein standards (Bio-Rad, CA, USA) were electrophoresed in parallel with the focused proteins.

Gels were fixed in 10% (v/v) methanol and 7% (v/v) glacial acetic acid for 1 hr prior to staining with Coomassie brilliant blue R-250 [15]; Silver stain as described by [16] or SYBRO Ruby according to the manufacturer’s instructions (Bio-Rad). High-resolution digital images of Coomassie Blue or Silver-stained gels were obtained using a scanner (Agfa, USA). Fluorescent images were obtained using a DyNA Light UV transilluminator (Labnet International Inc. Woodbridge, NJ, USA). Two-dimensional SDS-PAGE gel analyses were accomplished with the aid of PDQuest^TM^ 2-D Analysis Software (BIO-RAD).

As far as maximal reproducibility of 2D-PAGE protein profiles is concerned, the same stock and working solutions, protocols for sample preparation, and downstream procedures for the entire study were employed. Furthermore, simultaneous electrophoresis of batches of gels was performed exploiting multiple, vertical 2D-PAGE systems. All experiments were performed in triplicate. Prior to carrying out comparisons between different gels, polypeptide spots were normalized using the previous software. The density of polypeptide spots was expressed as the mean of three independent gels ± standard deviation (SD).

#### “In-gel” tryptic digestion and peptide extraction

Tryptic in-gel digestion was carried out as described earlier [17, 18] with some modifications: selected polypeptide band from 3 replicate gels was excised from stained 1-D gels. Briefly, Coomassie-stained polypeptide band was homogenized, destained, and washed with 100 mM ammonium bicarbonate, 100 mM ammonium bicarbonate/50% acetonitrile, and 100% acetonitrile in a consecutive manner. The isolated polypeptides were then dried and trypsinized in 50 μL of a 12 ng/μl sequence-grade modified porcine trypsin (Promega, Madison, USA) in 50 mM ammonium bicarbonate, pH 8.6 at 37°C overnight. The extracted tryptic peptide mixtures were concentrated and purified using a Speedvac and C18 Zip Tips according to manufacturer’s instructions (Millipore, Billerica, Massachusetts, USA) in corresponding order, and analyzed by matrix-assisted laser desorption ionization—time of flight (MALDI-TOF) mass spectrometry (PerSeptive Biosystems, Framingham, Massachusetts, USA).

#### MALDI-TOF mass spectrometric analysis and database search

The peptide mass fingerprint (PMF) data were used as a unique identifier of the protein to search the following databases: Mascot (http://www.matrixscience.com), ToxoDB (www.toxodb.org/toxo), Eukaryotic Pathogens Database Resources (http://eupathdb.org/eupathdb), and the NCBI nr database (ftp://ftp.ncbi.nih.gov/blast/db/FAST/nr.gz).

### Calculation of Genetic Distance based on Parasite Strain Proteomes

The genetic distance between the two strains was determined according to [2, 19].

Formula used:

$${\text{D}}_{\text{M}6,\;\text{GS}}=1\;\text{-}\;{\text{N}}_{\text{M}6,\;\text{GS}}÷[({\text{N}}_{\text{M}6}+{\text{N}}_{\text{GS}})\;\text{-}\;{\text{N}}_{\text{M}6,\;\text{GS}}]$$

where D_M6, GS_ = the genetic distance between *E*. *maxima* strain GS and M6;

N_M6, GS_ = the number of polypeptide spots shared by strain GS and M6 of *E*. *maxima;*

N_M6_ = the number of polypeptide spots in strain M6 of *E*. *maxima;* N_GS_ = the number of polypeptide spots in strain GS of *E*. *maxima*.

## Results

### One-dimensional polyacrylamide gel electrophoresis

#### The polypeptide band comparison between the sporozoites of *E*. *maxima* strains: GS and M6 exploiting PBS-lysis buffer (pH 7.2)

**Fig 1:**
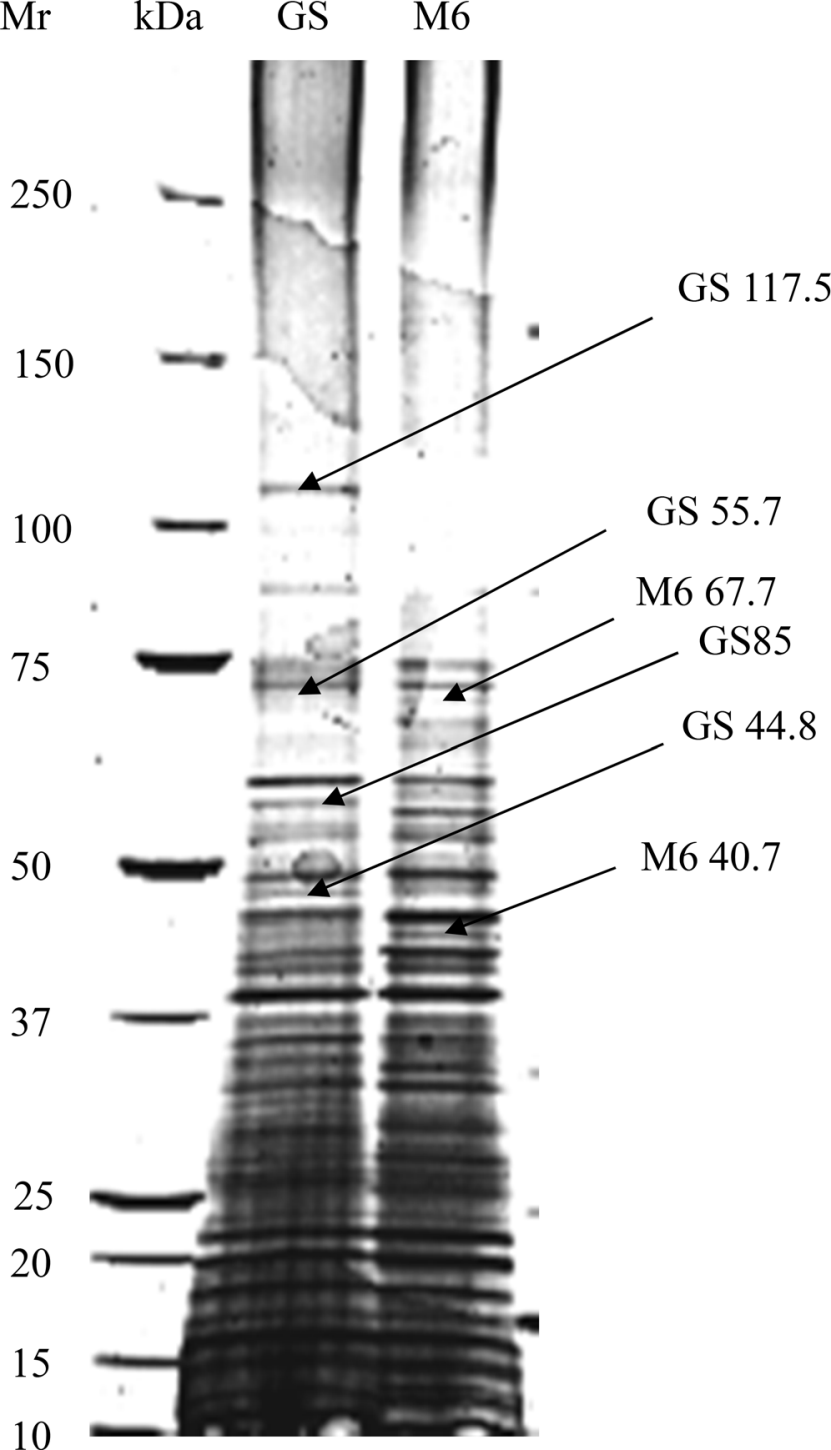
Coomassie blue-stained gradient sodium dodecyl sulphate-PAGE (5–20%) of the whole sporozoite polypeptide bands of *E*. *maxima* Guelph (Lane 2) and M6 (Lane 3) strains using PBS-lysis buffer (pH 7.2). Molecular weight standard, which ranges from 10 to 250 kD_a_ from the bottom to the top co-electrophoresed in the same gel as presented in lane 1. Arrows with M_r_ in lane 2 and 3 indicate the differences.

Coomassie blue-stained polypeptide bands of *E*. *maxima* GS and *E*.*maxima* M6 sporozoites separated by 1D-SDS-PAGE are set out in Fig 1. The Coomassie blue stained gel of *E*. *maxima* M6 sporozoite showed remarkably similar collection of polypeptides over the broad range of molecular weights with the following exceptions: polypeptide bands M6 40.7 and M6 67.7 kDa were absent or barely detectable in *E*. *maxima* strain GS (Fig 1). Furthermore, *E*. *maxima* GS bands; GS 117.5 is absent in *E*. *maxima* strain M6. However, GS 55.7 and GS 44.8 kDa were highly detectable in *E*. *maxima;* GS strain compared with those of *E*. *maxima* strain M6.

#### The polypeptide band comparison between *E*. *maxima* strains: GS and M6 sporozoites employing Tris-lysis buffer (pH 9.6)

**Fig 2:**
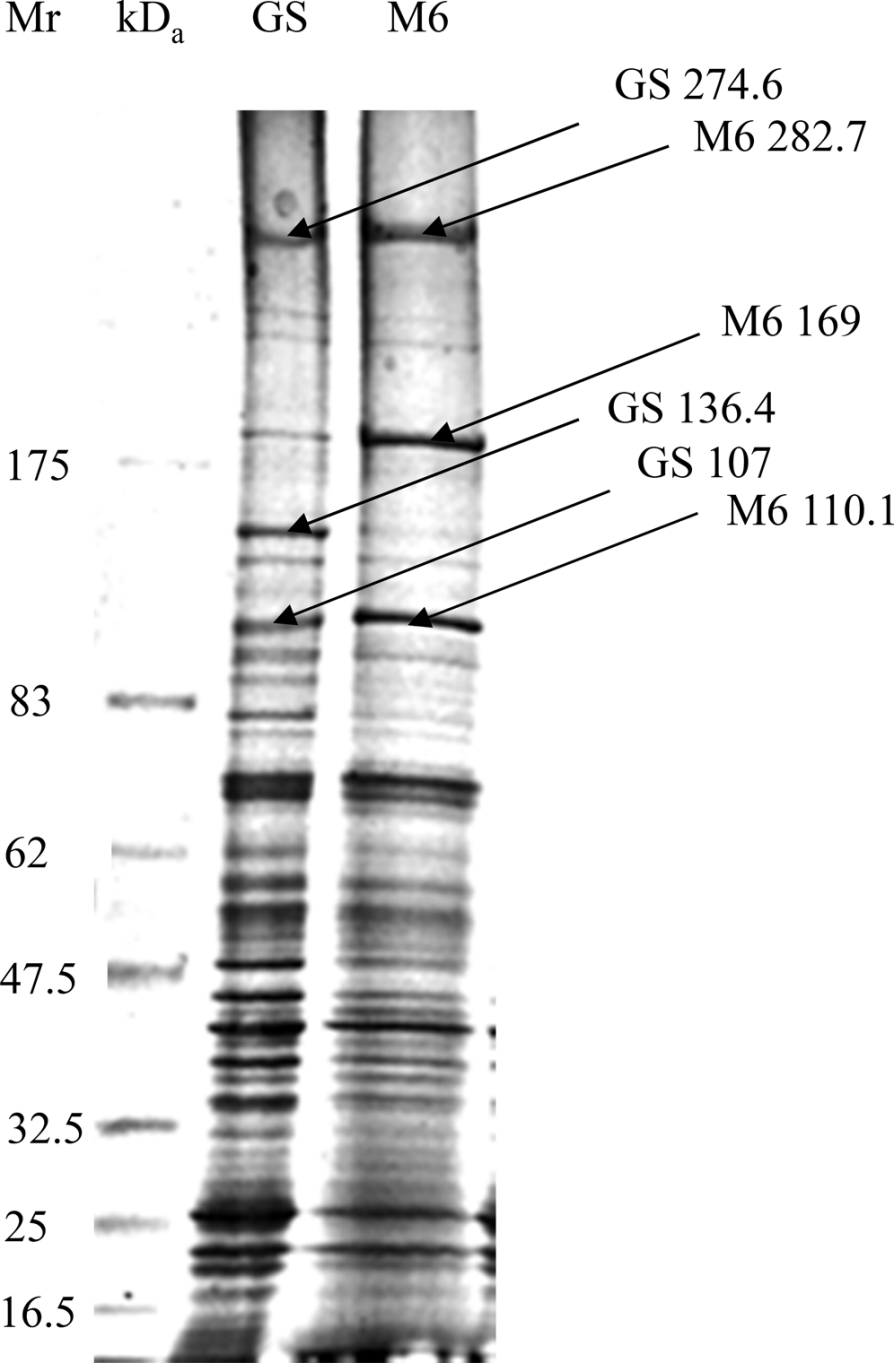
Coomassie blue-stained gradient SDS-slab gel (5–20%) of the sporozoites of *E*. *maxima* Guelph and M6 strains using Tris-lysis buffer (pH 9.6). Lane 1: Molecular weight standard ranges from 16 to 175 kDa from the bottom to the top. Lane 2: *E*. *maxima* GS Lane 3: *E*. *maxima* M6. Arrows in 2 and 3 indicate the differences

The polypeptide bands of *E*. *maxima* strains: GS and M6 sporozoites were separated on the basis of molecular weights using 5–20% gradient SDS-PAGE. The polypeptide bands consistently showed some differences in the electrophoretic mobility after Coomassie blue staining. The first dimensional-SDS-PAGE staining pattern disclosed that the GS 274.6 and GS 107 polypeptide bands had a faster electrophoretic mobility than M6 282.7 and M6 110.1 respectively (Fig 2). It was also shown that the polypeptide bands; GS 136.4 and M6 169 were intensely stained utilizing Coomassie blue stain.

#### Mass spectrometric identification of proteins

**Fig 3:**
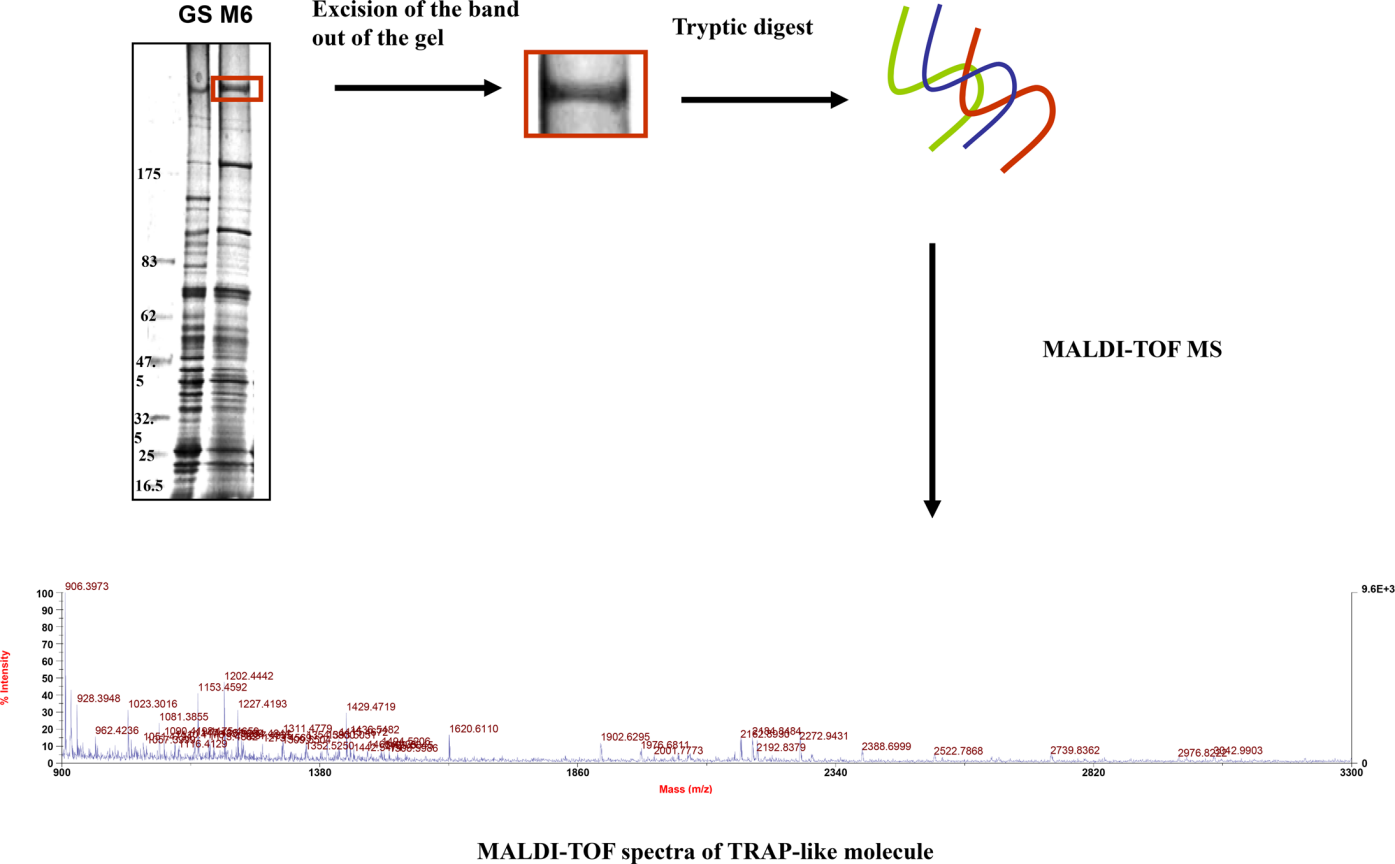
Scheme showing the procedure of peptide mass fingerprinting.

The separation of complex protein mixtures by one-dimensional polyacrylamide gel electrophoresis (1-D PAGE) in combination with MALDI-TOF analysis followed by peptide mass database searches was used for protein identification. The in-gel digestion and MADI-TOF analysis for the identification of M6 272.5 band is outlined in (Fig 3).

The results, as shown in Fig 3, involve the list of masses obtained from M6 272.5 band digest served as the focal point for identifying and characterizing primary structural features in the M6 272.5 band. Experimentally determined masses were used with MASCOT peptide mass fingerprinting (PMF) database searching to match theoretical peptide masses and to predict M6 272.5 band identities (Fig 3). 46 peptides were used for a database search, which identified the M6 272.5 band as TFP250 with 88 score.

The nominal mass (Mr) and the calculated pI were 262860 and 4.19 respectively.

#### The polypeptide spot comparison between *E*. *maxima* strains: GS and M6 sporozoites

**Fig 4:**
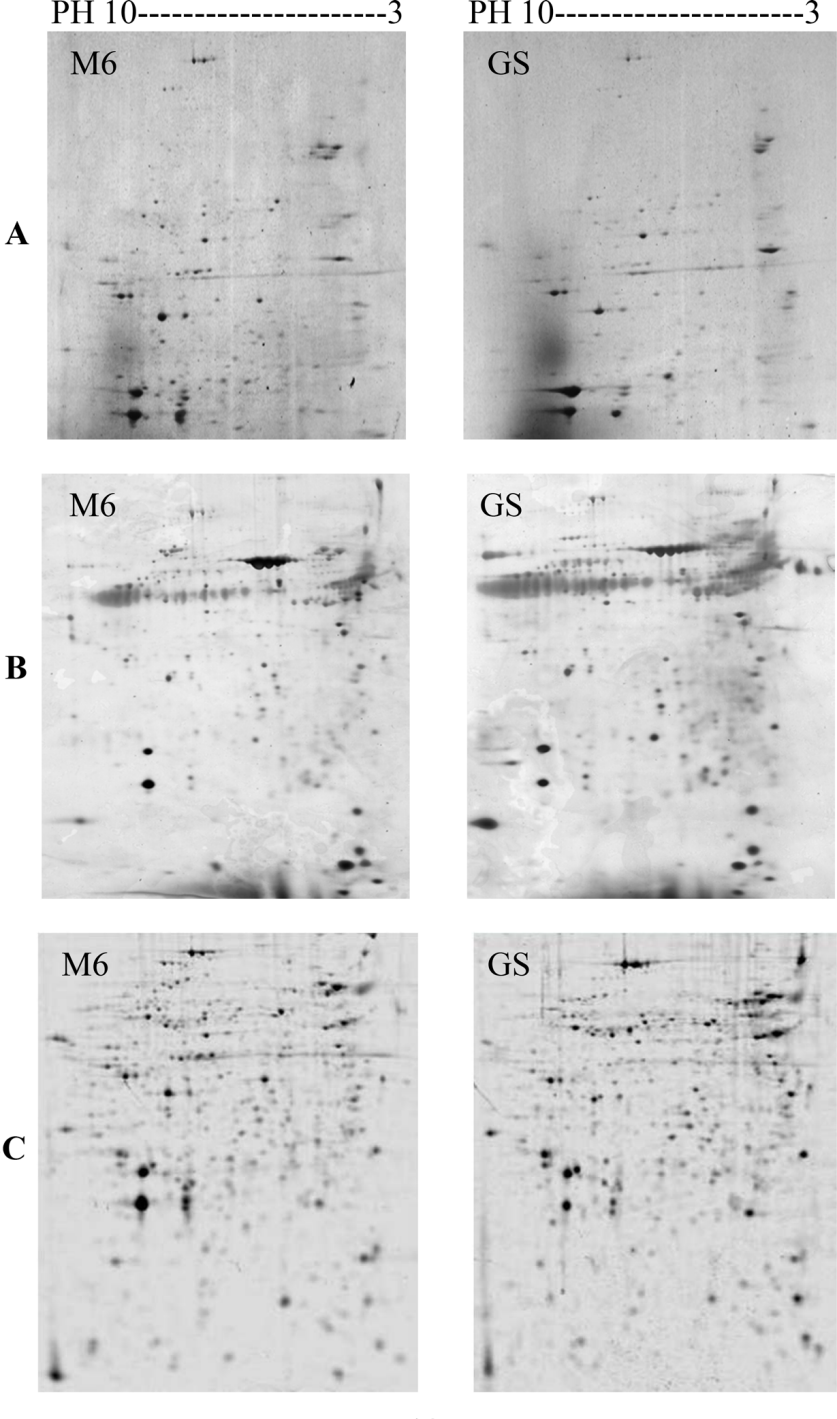
Two-dimensional reference maps of the sporozoite polypeptide spots of *E*. *maxima* M6 and Guelph strains using Tris-lysis buffer (pH 9.6). The total sporozoite proteins were resolved by isoelectric focusing (IEF) and separated across the pH range 3–10, 12.5% acrylamide gel. A. The polypeptide spots were visualized with Coomassie brilliant blue R-250. B. The polypeptide spots were visualized with Silver stain. C. The polypeptide spots were visualized with SYPRO Ruby stain.

The large-scale of the *E*. *maxima* strains: M6 and GS proteome was characterized by two-dimensional polyacrylamide gel electrophoresis (2D-PAGE). The sporozoite fractionation of *E*. *maxima* strains: M6 and GS proteins was done using the immobilized pH gradient (IPG) 3–10 non-linear gel strip according to the isoelectric point(1D), and then the sporozoite polypeptide spots were resolved according to their molecular weights (2D). The relative sensitivity of 2D-PAGE was determined after staining of the polypeptide spots resolved on the gels by different staining solutions. A small number of polypeptide spots in the case of Coomassie brilliant blue R-250 and Silver compared to SYPRO Ruby stain (Fig 4A–4C). The number of separated polypeptide spots of *E*. *maxima* strains sporozoite proteins were detected by SYBRO Ruby over the pH range of 3–10 nonlinear is shown in Table 1.

**Table 1:** The variables polypeptide spots (i.e. shared and unique polypeptide spots).

*E*. *maxima*	Guelph strain (GS)	M6 strain
Total number of resolved spots	696	694
Number of different resolved spots	69*	67*

*Unique polypeptide spot

### Genetic divergence between the two strains GS and M6 of *E*. *maxima*

Based on the polypeptide spots identified in paired 2-D gels stained with SYPRO Ruby, the genetic distance was calculated as follows:

$${\text{D}}_{\text{M}6,\;\text{GS}}=1\;\text{-}\;{\text{N}}_{\text{M}6,\;\text{GS}}÷[({\text{N}}_{\text{M}6}+{\text{N}}_{\text{GS}})\;\text{-}\;{\text{N}}_{\text{M}6,\;\text{GS}}]$$

$${\text{D}}_{\text{M}6,\;\text{GS}}=0.1782438$$

Thus, D_M6, GS_ is a measure of the genetic distance between the two strains with numbers approaching zero indicating decreasing distance between organisms. Therefore, the genetic similarity is: 1- D_M6, GS_ = 1–0.1782438 = 0.8217562.

**Fig 5:**
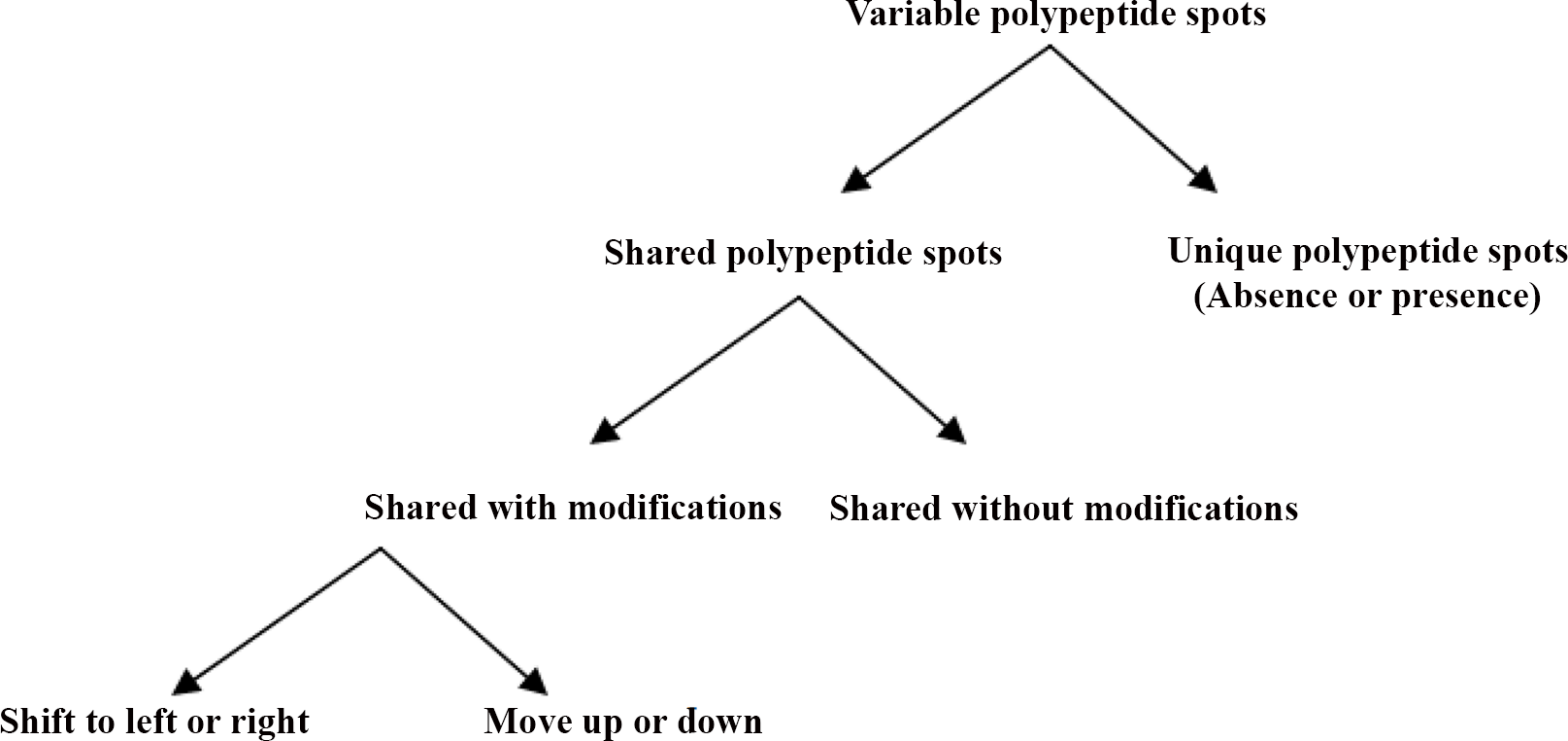
Variable polypeptide spot flow chart.

**Fig 6:**
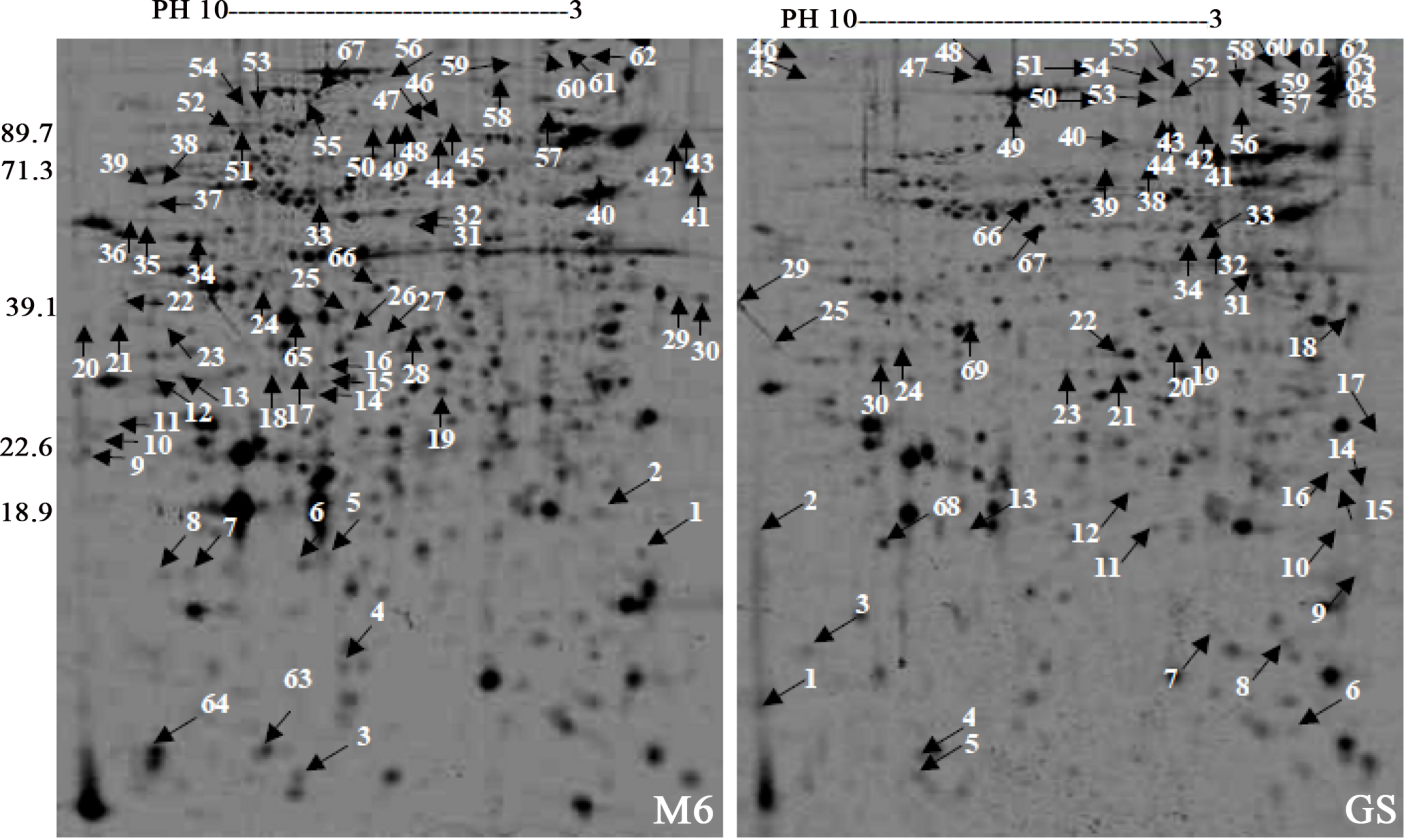
Annotated polypeptide spot nos. using arrows and M_r_ of the two-dimensional reference maps of *E*. *maxima* M6 and Guelph strains using Tris-lysis buffer (pH 9.6). The total sporozoite proteins were resolved by isoelectric focusing (IEF) and separated across the pH range 3–10, 12.5% acrylamide gel. The polypeptide spots were visualized with SYPRO Ruby stain.

**Fig 7:**
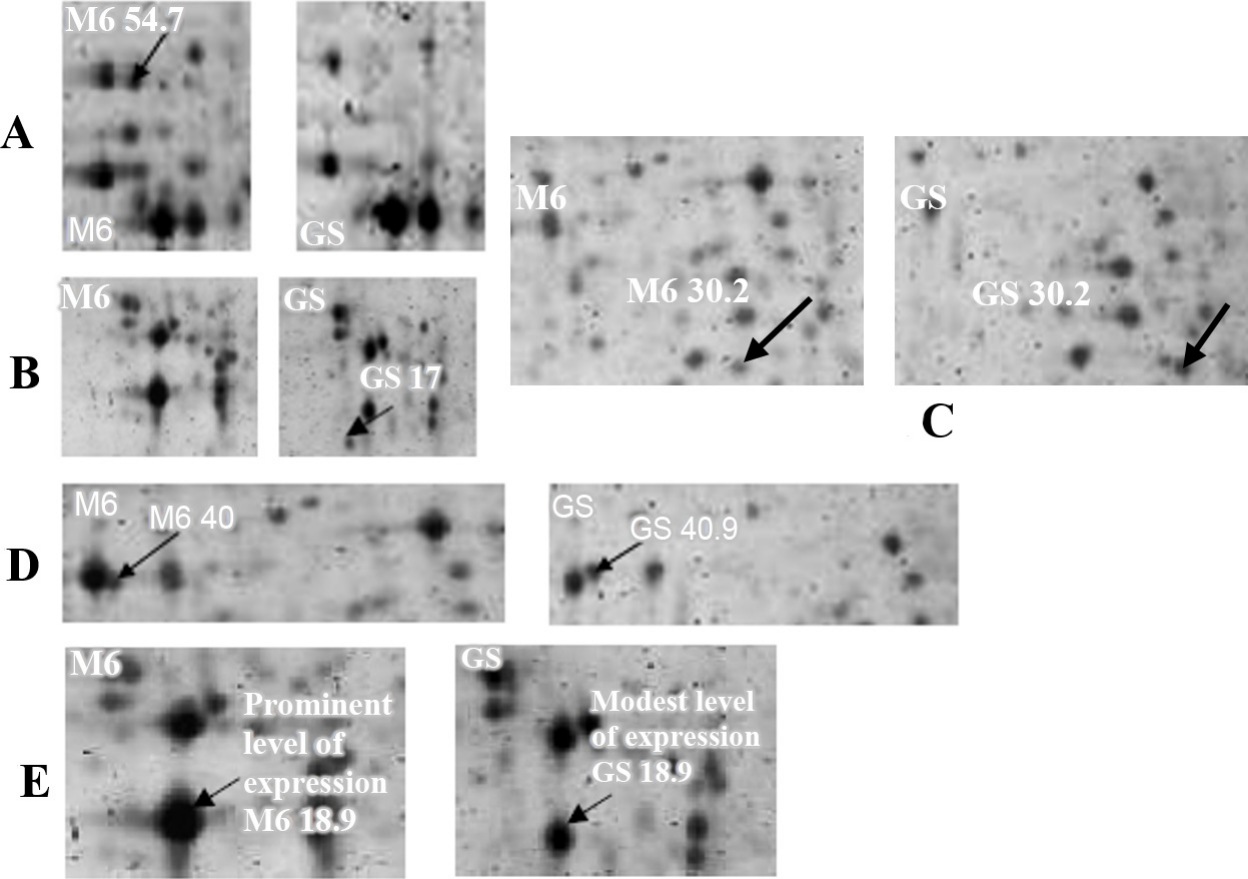
Illustration of variable polypeptide spots. A. Region in the gel containing polypeptide spot that is in the reference map of *E*. *maxima* M6 and not in the reference map of *E*. *maxima* GS. B. Section of the gel containing polypeptide spot that is in the reference map of *E*. *maxima* GS and not in the reference map of *E*. *maxima* M6. C. Regions in the representative gel reference maps of two stains of *E*. *maxima* M6 and GS display shift to left or right of the two polypeptide spots; M6 30.2 and GS 30.2 as indicated by arrows. D. Regions in the representative gel reference maps of two stains of *E*. *maxima* M6 and GS show move up and down of the two polypeptide spots; M6 40 and GS 40.9 as indicated by arrows. E. Parts of the representative gel reference maps of two stains of *E*. *maxima* M6 and GS display overexpression of the two polypeptide spots as indicated by arrows.

The genetic distance is nearer to zero indicating that the close resemblance of the two strains of the same species. The variable polypeptide spots could be divided according to the following flow chart as shown in Fig 5 and the illustrated Figs 6 and 7A–7D. The results of over-expression of the same polypeptide spots are shown in Fig 7E.

By comparing the two strains GS and M6 of *E*. *maxima*, the unique polypeptide spots (present in one strain and not in the other strain) can be identified based upon the following criteria: isoelectric point that covers the wide range of pH 3–10, molecular weight and the relative intensities (Tables 2 and 3).

**Table 2:** Isoelectric point (pI), molecular weight (M_r_), and quantity of polypeptide spots of 2-DE separated *E*. *maxima* M6 strain sporozoite proteins (Fig 6).

Spot No.	pI	M_r_ (KD_a_)	Quantity	Spot No.	pI	M_r_ (KD_a_)	Quantity
1	3.8	15.9	1.2×10^3^±1.53×10^3^	35	9	59.4	3.9×10^3^±0.17×10^3^
2	4.2	18.8	15.98×10^3^±2.52×10^3^	36	9.3	59.4	2.4×10^3^±0.05×10^3^
3	6.5	6.7	18.5×10^3^±3.58×10^3^	37	9	62.9	11.3×10^3^±0.2×10^3^
4	6	10.3	12.3×10^3^±3×10^3^	38	8.7	67.9	3.28×10^3^±0.07×10^3^
5	6.2	15.7	22.6×10^3^±4.2×10^3^	39	9	67.9	1.29×10^3^±0.04×10^3^
6	6.4	15.2	20.67×10^3^±4×10^3^	40	4.2	69.4	6.23×10^3^±0.03×10^3^
7	8.1	14.8	9.3×10^3^±3.4×10^3^	41	3.3	69.8	12.78×10^3^±0.03×10^3^
8	8.7	14.7	8.95×10^3^±2.1×10^3^	42	3.5	79.8	3.65×10^3^±0.01×10^3^
9	10	27.1	14.9×10^3^±3.5×10^3^	43	3.6	84.2	3.97×10^3^±0.07×10^3^
10	10	27.2	8.5×10^3^±1.81×10^3^	44	5.7	82.4	5.6×10^3^±0.35×10^3^
11	10	20.2	5.7×10^3^±1×10^3^	45	5.6	84.6	3.57×10^3^±0.08×10^3^
12	8.9	32.1	8.7×10^3^±2.4×10^3^	46	5.7	84.6	3.65×10^3^±0.1×10^3^
13	8.3	32.1	13.4×10^3^±1.5×10^3^	47	7.9	84.6	5.68×10^3^±0.05×10^3^
14	6.3	29.8	5.5×10^3^±2.9×10^3^	48	5.7	64.6	1.1×10^3^±0.09×10^3^
15	6.2	31.1	5.8×10^3^±2.2×10^3^	49	5.8	84.6	1.1×10^3^±0.05×10^3^
16	6.2	32.8	6.65×10^3^±3×10^3^	50	5.8	84.6	3.8×10^3^±0.26×10^3^
17	6.4	32.8	4.96×10^3^±0.4×10^3^	51	7.7	82.8	5.7×10^3^±0.27×10^3^
18	6.7	32.5	8.4×10^3^±0.13×10^3^	52	7.9	82.8	6.9×10^3^±0.06×10^3^
19	5.5	30.2	18.9×10^3^±0.26×10^3^	53	7.5	90.1	3.6×10^3^±0.198×10^3^
20	8.9	39.7	1.8×10^3^±0.2×10^3^	54	7.4	90.1	4.9×10^3^±0.2×10^3^
21	9.5	39.7	4.44×10^3^±0.096×10^3^	55	6.4	94.2	4.7×10^3^±0.2×10^3^
22	9.5	42.2	4.3×10^3^±0.05×10^3^	56	5.9	102.6	3.4×10^3^±0.15×10^3^
23	8.7	39.2	4.72×10^3^±0.072×10^3^	57	5	91.4	4.7×10^3^±0.26×10^3^
24	8.5	44.7	5.26×10^3^±0.04×10^3^	58	5.2	99.6	3.7×10^3^±0.25×10^3^
25	6	40.9	5.97×10^3^±0.082×10^3^	59	5.2	103.8	2.4×10^3^±0.14×10^3^
26	6	37.2	13.6×10^3^±0.15×10^3^	60	5	106.5	3.97×10^3^±0.05×10^3^
27	5.8	37.5	5.96×10^3^±0.15×10^3^	61	4.6	106.5	21.4×10^3^±0.05×10^3^
28	5.6	37.7	13.5×10^3^±0.09×10^3^	62	4.4	106.1	11.74×10^3^±0.04×10^3^
29	3.55	44.7	2.7×10^3^±0.2×10^3^	63	6.8	7.5	40.7×10^3^±0.27×10^3^
30	3.3	43.5	11.6×10^3^±0.35×10^3^	64	8.9	7.1	71.2×10^3^±0.17×10^3^
31	5.6	57.6	4.28×10^3^±0.08×10^3^	65	6.6	40	27.3×10^3^±0.13×10^3^
32	5.6	59.4	3.9×10^3^±0.2×10^3^	66	5.8	45.4	17.8×10^3^±0.2×10^3^
33	6.2	62.6	3.6×10^3^±0.17×10^3^	67	6.3	96.7	7.5×10^3^±0.15×10^3^
34	8	54.7	18.2×10^3^±0.09×10^3^				

**Table 3:** Isoelectric point (pI), molecular weight (M_r_), and quantity of polypeptide spots of 2-DE separated *E*. *maxima* GS strain sporozoite proteins (Fig 6).

Spot No.	pI	M_r_ (KD_a_)	Quantity	Spot No.	pI	M_r_ (KD_a_)	Quantity
1	10	8.7	29.8×10^3^±0.26×10^3^	36	3.3	61.4	1.1×10^3^±0.01×10^3^
2	10	17.6	8.4×10^3^±0.05×10^3^	37	3.3	65.8	0.9×10^3^±0.09×10^3^
3	9.6	10.9	8.2×10^3^±0.35×10^3^	38	7.8	78.7	1.2×10^3^±0.05×10^3^
4	6.9	7.2	14.6×10^3^±0.35×10^3^	39	7.9	78.7	1.5×10^3^±0.01×10^3^
5	5.1	6.7	22.4×10^3^±0.2×10^3^	40	7.8	84.5	3×10^3^±0.13×10^3^
6	4.4	8.1	11.1×10^3^±0.04×10^3^	41	5.1	86.7	49.9×10^3^±0.53×10^3^
7	7.4	12.1	6.8×10^3^±0.105×10^3^	42	5.2	90.3	3.1×10^3^±0.05×10^3^
8	4.4	11.6	9.1×10^3^±0.048×10^3^	43	5.4	91.3	1.51×10^3^±0.008×10^3^
9	3.6	14.9	14.2×10^3^±0.18×10^3^	44	7.7	91.3	2.1×10^3^±0.025×10^3^
10	3.7	18.1	4.3×10^3^±0.095×10^3^	45	9.6	103.2	1.2×10^3^±0.04×10^3^
11	5.3	18.2	9.6×10^3^±0.33×10^3^	46	9.7	114.2	1.9×10^3^±0.087×10^3^
12	5.4	20.8	6×10^3^±0.5×10^3^	47	6.4	109.5	2.7×10^3^±0.05×10^3^
13	6.4	17.8	8.3×10^3^±0.062×10^3^	48	6.3	109.2	2.6×10^3^±0.15×10^3^
14	3.6	21.2	7.4×10^3^±0.16×10^3^	49	6.2	96.9	5.2×10^3^±0.02×10^3^
15	3.7	21.6	2.1×10^3^±0.06×10^3^	50	5.7	99.4	3.4×10^3^±0.044×10^3^
16	3.7	23	3.3×10^3^±0.09×10^3^	51	5.7	114.2	1.2×10^3^±0.025×10^3^
17	3.5	25.4	2.8×10^3^±0.2×10^3^	52	7.7	99.4	1.9×10^3^±0.07×10^3^
18	3.6	42.1	17×10^3^±0.95×10^3^	53	5.2	103.9	30.3×10^3^±0.09×10^3^
19	5.2	38.5	2.4×10^3^±0.05×10^3^	54	5.2	108.7	4.8×10^3^±0.2×10^3^
20	5.2	37.7	2.5×10^3^±0.25×10^3^	55	5.1	108.7	2.9×10^3^±0.05×10^3^
21	5.6	33.2	10.3×10^3^±0.09×10^3^	56	4.8	98.4	2.1×10^3^±0.05×10^3^
22	5.6	36.3	5.6×10^3^±0.13×10^3^	57	4.7	71.3	4.2×10^3^±0.06×10^3^
23	5.8	33.5	8.5×10^3^±0.44×10^3^	58	4.9	104.4	3.8×10^3^±0.06×10^3^
24	7.3	37.1	12.16×10^3^±×10^3^	59	4.7	104.4	3.2×10^3^±0.046×10^3^
25	9.9	36.9	1.1×10^3^±0.05×10^3^	60	4.7	113.6	5.4×10^3^±0.087×10^3^
26	10	39.7	2.7×10^3^±0.26×10^3^	61	4.2	112.5	1.7×10^3^±0.15×10^3^
27	10	40.3	2×10^3^±0.18×10^3^	62	4.1	113.6	1.2×10^3^±0.036×10^3^
28	10	41.5	1.6×10^3^±0.025×10^3^	63	4.1	108.7	2.5×10^3^±0.1×10^3^
29	10	43.4	4.3×10^3^±0.09×10^3^	64	4.1	105.2	2.4×10^3^±0.098×10^3^
30	7.6	35	17.9×10^3^±0.17×10^3^	65	4.1	98.7	1.5×10^3^±0.086×10^3^
31	4.8	36.6	2.2×10^3^±0.1×10^3^	66	6	64.7	4.9×10^3^±0.1×10^3^
32	5	57.1	2.7×10^3^±0.15×10^3^	67	5.9	59.8	2.1×10^3^±0.02×10^3^
33	5.1	57.1	7.5×10^3^±0.09×10^3^	68	7.6	17	2.8×10^3^±0.26×10^3^
34	5.2	57.1	2.3×10^3^±0.1×10^3^	69	6.6	40.9	2.2×10^3^±0.087×10^3^
35	3.3	56.7	1.3×10^3^±0.05×10^3^				

## Discussion

This study pertains to analysis of the protein profile of two immunologically distinct strains of *E*. *maxima* by 2D gel electrophoresis. Although not observed in previous 1-D SDS-PAGE analyses [12], this study was able to distinguish between the sporozoite proteins of two strains of *E*. *maxima* GS and M6 utilizing 1-D SDS-PAGE. In particular, polypeptide bands; GS 117.5 was not apparent in the protein profile of *E*. *maxima* M6 lysed using a pH 7.2 lysis buffer. Polypeptide bands; M6 40.7 and M6 67.7 kDa were absent or barely detectable in *E*. *maxima* GS using the same lysis buffer. However, GS 55.7 and GS 44.8 kDa were highly detectable in *E*. *maxima;* GS strain compared with that of *E*. *maxima* M6 using macro-scale SDS-PAGE technique and pH 7.2 as a lysis buffer. By using the more alkaline lysis buffer (Tris pH 9.6); this study was able to detect some more subtle differences in the electrophoretic mobility between the strains such as bands GS 274.6 and GS 107 demonstrating a faster electrical mobility than M6 282.7 and M6 110.1 bands, respectively. The electrophoretic mobility between the two aforementioned strains was observed: GS 274.6 polypeptide band has a faster electrical mobility than M6 282.7. The excised band (i.e. M6 272.5) were digested by trypsin and then the tryptic digest was analysed with Matrix-assisted laser desorption ionization time of flight (MALDI-TOF) analysis of the M6 272.5 band tentatively identified this polypeptide band as a thrombospondin-related adhesive protein (TRAP)-like molecule that had been characterized previously from different strain of *Eimeria maxima* (EmTFP250; *Eimeria maxima* TRAP Family Protein 250 kDa). The findings of the current study are consistent with those of [20, 21] who detected the same molecule; EmTFP250 of the asexual stage antigen of *Eimeria maxima*. The difference in the molecular weight between the two bands (i.e. GS 267.1 and M6 272.5) that was detected at 1-D SDS-PAGE level may be due to differences in the primary sequence of the molecules in the N-terminal regions of the ectodomain of the molecule and/or post-translational modifications to one or either of these molecules (unpublished data).

Therefore, we are forced to elaborate the findings of this study into vaccine which comprises TRAP polypeptides, CD 154 polypeptide, and *Salmonella entritidis*. We accredit the reader to our published patent for detailed information [22].

In addition to differences in both molecular weight and the electrophoretic mobility, this study explored differences in the intensity of polypeptide bands as a measure of differences in the expressed proteins of these two strains of *E*. *maxima*. For example, polypeptide bands; GS 136.4 and M6 169 were intensely stained with Coomassie blue stain. This results contrast with that of [12] who mentioned that no differences were observed by 1-D SDS-PAGE. The close proximity of molecular weight bands that bear the same charge and the possibility for the presence of more than one different polypeptide in the bands in the electrophoretic profile of the two strains resolved by 1-D SDS-PAGE technique led to the use of 2D SDS-PAGE with its intrinsically higher resolving power. This ability to separate proteins on the basis of two different parameters (i.e. charge and molecular weight) was exploited first by [1]. Sensitive visualization of polypeptide spots over a wide linear range is a crucial step for quantification of gene expression at the protein level. Comparison of the stained gels by different stains showed that the SYPRO Ruby fluorescent stain was the more sensitive stain in detection of polypeptide spots than Coomassie blue and Silver stain as had been observed previously. SYPRO Ruby is more convenient, reproducible and an endpoint procedure. This result is in agreement with [17] who reported that Silver stain was not an end point. By combining a large format 2-D electropherogram together with a more sensitive stain, this study was able to finally demonstrate phenotypic differences between these two immunovariant strains that can potentially explain the lack of cross-reactivity between these parasites in immunocompetent chickens. By combining high resolution 2-D SDS-PAGE with sophisticated image analysis; differences between the strains both in the presence and absence of spots (67unique spots in M6 and an additional 69 unique spots in GS), as well as in the relative expression of various polypeptide spots shared by both strains were detected. In addition to the differences in the level of some polypeptide spots expression (i.e. high expression level versus low level of expression) which is probably due to codon bias; the presence of several copies of the gene that is responsible for the gene product or the presence of mixture of different polypeptides; the sporozoite 2D electropherogram of the *E*. *maxima* strains obtained from a consensus polypeptide array of around 10 gels displayed a large number of the variable polypeptide spots. This result is consistent with [2] who was able to produce fingerprint maps to differentiate between the 7 species of *Eimeria*. The variable polypeptide spots could be divided into unique (presence/absence) and shared polypeptide spots. The latter could be categorized into shared with and without post-translational modifications. The post-translational modifications could be further sorted into move up or move down, such as GS 29.3 and M6 29 or shift to left or to the right, such as GS 20 and M6 19.8.

Of 696 *E*. *maxima* M6 and 694 *E*. *maxima* GS polypeptide spots resolved by two dimensional polyacrylamide gel electrophoresis and analyzed by PDQuest^TM^ 2-D Analysis Software; only a few differences 69 and 67 unique polypeptide spots were detected for *E*. *maxima* M6 and 694 *E*. *maxima* GS respectively. Relatedly, a high-resolution 2-DE gel separation over pH ranges 4–7 and 3–10 was also exploited to resolve 460 and 600 polypeptides spots from *Eimeria tenella* sporozoite using 4–7 and 3–10 pH strips respectively [3], and in a separate study, a total of 845 polypeptides spots in *Eimeria tenella* sporozoite proteome was identified [23].

A reasonable approach to tackle the lackability of cross-species and even cross-strain protection could be to hypothesize the following hypotheses to unravel this limitation:

1 Shared modified- polypeptide spots in the pair of *E*. *maxima* strain

The selection pressure elicited by the host on the parasite leads to the emergence of shared modified polypeptide spots, for example GS P20 and M6 P19.8 appear to be post-translation modification and are probably not related to genomic changes (i.e. at the level of mRNA). According to this hypothesis, these shared modified- polypeptide spots GS P20 and P19.8, could represent the business end of an invasive stage or hidden antigens that are used to evade the host immune system prior to establishment of the parasite or to evade the immune system after establishment, respectively. Mass spectrometry and N-terminal amino acid sequence should be done to determine the sequence of these interesting molecules so that their likely identity can be determined.

2 In relation to the spots that are present in one line and not in the other, the protective capability of the unique spots (i.e. presence/absence) in the immunovariant lines can be tested by the following approach:
  2.1 Generation of attenuated lines (generally an abbreviated life cycle that results from deletion of one or more asexual endogenous cycles) from the two parent lines.
  2.2 1D-and 2-D-PAGE of the generated precocious lines should be examined to compare between precocious lines and the respective parental lines.

By comparing 1D- and 2-D-PAGE of the precocious (abbreviated life cycle) lines with the parent lines, the polypeptide spots, which disappear, can be safely excluded from the collection of previously identified unique polypeptide spots (i.e. unique for each line) because precocious lines protect birds against challenge by the parent line of parasite.

3- T-cell and B-cell epitopes.

B-cell epitopes are usually considered irrelevant with respect to the protective adaptive immune response because bursectimized birds are still protected against homologous challenge infections. Therefore, unique spots on 2-D gels (from either parent or precocious lines as appropriate) will be examined for their ability to elicit T-cell responses (e.g. IFN-gamma and IL-4 ELISPOT to differentiate Th1 versus Th2 biases responses, respectively). Taken together, these findings and hypotheses may lead to novel vaccine target in the form of multi-component, multi-stage, multi-immunovariant strains, multi-species subunit vaccine, and diagnostic probe for *E*. *maxima*.
